# Comparison of oxidative stress markers in HIV-infected patients on efavirenz or atazanavir/ritonavir-based therapy

**DOI:** 10.7448/IAS.17.4.19544

**Published:** 2014-11-02

**Authors:** Vicente Estrada, Susana Monge, Dulcenombre Gómez-Garre, Paz Sobrino, Juan Berenguer, Jose Ignacio Bernardino, Jesús Santos, Ana Moreno Zamora, Esteban Martínez, Jose Ramón Blanco

**Affiliations:** 1 Infectious Diseases, Hospital Clinico San Carlos, Madrid, Spain; 2 Instituto de Salud Carlos III, Centro Nacional de Epidemiología, Madrid, Spain; 3 Vascular Biology Lab, Hospital Clinico San Carlos, Madrid, Spain; 4 Infectious Diseases, Hospital General Gregorio Marañón, Madrid, Spain; 5 Infectious Diseases, Hospital La Paz, Madrid, Spain; 6 Infectious Diseases, Hospital Virgen de la Victoria, Malaga, Spain; 7 Infectious Diseases, Hospital Ramon y Cajal, Madrid, Spain; 8 Infectious Diseases, Hospital Clinic, Barcelona, Spain; 9 Infectious Diseases, Hospital San Pedro-CIBIR, Logroño, Spain

## Abstract

**Introduction:**

Chronic low-grade inflammation and immune activation may persist in HIV patients despite effective antiretroviral therapy (ART). These abnormalities are associated with increased oxidative stress (OS). Bilirubin (BR) may have a beneficial role in counteracting OS. Atazanavir (ATV) inhibits UGT1A1, thus increasing unconjugated BR levels, a distinctive feature of this drug. We compared changes in OS markers in HIV patients on ATV/r versus efavirenz (EFV)-based first-line therapies.

**Materials and Methods:**

Cohort of the Spanish Research Network (CoRIS) is a multicentre, open, prospective cohort of HIV-infected patients naïve to ART at entry and linked to a biobank. We identified hepatitis C virus/hepatitis B virus (HCV/HBV) negative patients who started first-line ART with either ATV/r or EFV, had a baseline biobank sample and a follow-up sample after at least nine months of ART while maintaining initial regimen and being virologically suppressed. Lipoprotein-associated Phospholipase A2 (Lp-PLA2), Myeloperoxidase (MPO) and Oxidized LDL (OxLDL) were measured in paired samples. Marker values at one year were interpolated from available data. Multiple imputations using chained equations were used to deal with missing values. Change in the OS markers was modelled using multiple linear regressions adjusting for baseline marker values and baseline confounders. Correlations between continuous variables were explored using Pearson's correlation tests.

**Results:**

145 patients (97 EFV; 48 ATV/r) were studied. Mean (SD) baseline values for OS markers in EFV and ATV/r groups were: Lp-PLA2 [142.2 (72.8) and 150.1 (92.8) ng/mL], MPO [74.3 (48.2) and 93.9 (64.3) µg/L] and OxLDL [76.3 (52.3) and 82.2 (54.4) µg/L]. After adjustment for baseline variables patients on ATV/r had a significant decrease in Lp-PLA2 (estimated difference −16.3 [CI 95%: −31.4, −1.25; p=0.03]) and a significantly lower increase in OxLDL (estimated difference −21.8 [−38.0, −5.6; p<0.01] relative to those on EFV, whereas no differences in MPO were found. Adjusted changes in BR were significantly higher for the ATV/r group (estimated difference 1.33 [1.03, 1.52; p<0.01]). Changes in BR and changes in OS markers were significantly correlated.

**Conclusions:**

In virologically suppressed patients on stable ART, OS was lower in ATV/r-based regimens compared to EFV. We hypothesize these changes could be in part attributable to increased BR plasma levels.

